# Quercetin induced ROS production triggers mitochondrial cell death of human embryonic stem cells

**DOI:** 10.18632/oncotarget.11070

**Published:** 2016-08-05

**Authors:** So-Yeon Kim, Ho-Chang Jeong, Soon-Ki Hong, Mi-Ok Lee, Seung-Ju Cho, Hyuk-Jin Cha

**Affiliations:** ^1^ College of Natural Sciences, Department of Life Sciences, Sogang University, Seoul 121–742, Korea; ^2^ Stem Cell Research Center, Korea Research Institute of Bioscience and Biotechnology (KRIBB), Daejeon 305–806, Korea

**Keywords:** quercetin, human embryonic stem cells (hESCs), reactive oxygen species (ROS), p53, cyclophilin D

## Abstract

Small molecules to selectively induce cell death of undifferentiated human pluripotent stem cells (hPSCs) have been developed with the aim of lowering the risk of teratoma formation during hPSC-based cell therapy. In this context, we have reported that Quercetin (QC) induces cell death selectively in hESCs via p53 mitochondrial localization. However, the detailed molecular mechanism by which hESCs undergo selective cell death induced by QC remains unclear.

Herein, we demonstrate that mitochondrial reactive oxygen species (ROS), strongly induced by QC in human embryonic stem cells (hESCs) but not in human dermal fibroblasts (hDFs), were responsible for QC-mediated hESC’s cell death. Increased p53 protein stability and subsequent mitochondrial localization by QC treatment triggered mitochondrial cell death only in hESCs. Of interest, peptidylprolyl isomerase D [*PPID*, also called cyclophilin D (CypD)], which functions in mitochondrial permeability transition and mitochondrial cell death, was highly expressed in hESCs. Inhibition of CypD by cyclosporine A (CsA) clearly inhibited the QC-mediated loss of mitochondrial membrane potential and mitochondrial cell death. These results suggest that p53 and CypD in the mitochondria are critical for the QC-mediated induction of cell death in hESCs.

## INTRODUCTION

Risk of teratoma formation from residual undifferentiated pluripotent stem cells (PSCs), due to high telomerase activity and active proliferation, has been considered to be one of the major roadblocks to the clinical application of hPSCs in cell therapy [1]. To resolve this problem, numerous attempts to selectively eliminate undifferentiated hPSCs, have been examined, including integration of suicide genes [2, 3], immunodepletion using antibodies [4], and selective induction of cell death using small molecules [5, 6]. Quercetin (QC), a natural flavonoid that has been studied in cancer due to its anti-proliferative and pro-apoptotic effects [7], was shown to induce selective cell death of residual undifferentiated PSCs through the mitochondrial apoptotic pathway in a p53-dependent manner [6]. However, the detailed molecular mechanism of QC-mediated cell death of PSCs remains unclear.

Although QC functions as either a pro-oxidant to produce reactive oxygen species (ROS) [8] or an anti-oxidant to protect from oxidative damage [9] depending on the cell model used, the cytotoxic activity of the pro-oxidant effect of QC occurs specifically in more aggressive cancer cells with active proliferation [10]. ROS are a type of radical anion generated by single-electron reduction of the oxygen during oxidative phosphorylation to generate ATP in mitochondria, which would be also relevant to chronological senescence [11]. Whereas a moderate ROS level is important for cell growth or differentiation, excess ROS can cause oxidative damage to DNA, proteins and lipids. Therefore, to maintain ROS homeostasis and protect against oxidative stress from ROS accumulation, cells develop a variety of anti-oxidants [12]. Similarly, although ROS play a critical role in ESC differentiation [13], high ROS stress causes ESCs to die [14] or lose pluripotency [15]. It is noteworthy that a set of anti-oxidants whose expression is regulated by p53 after oxidative stress [16], failed to be increased in murine ESCs during stress conditions, causing mitochondrial cell death due to mitochondrial localization of p53 [14].

ESCs are highly sensitive to genotoxic stresses including ROS [14] and chemotherapeutics [17, 18] due to BAX activity at the Golgi complex [19] and/or a lower mitochondrial cell death threshold than differentiated cells, referred to as ‘high mitochondrial priming’ [20]. High expression of pro-apoptotic factors in the mitochondria [6], high levels of cytoplasmic p53 [20] and/or rapid mitochondrial translocation of active BAX [19] account for rapid mitochondria dependent cell death of ESCs upon DNA damage stimuli [21].

Cyclophilin D (CypD), encoded by a gene called peptidylprolyl isomerase D (*PPID*), is expressed in the mitochondrial matrix [22] and has roles in the opening of the mitochondrial permeability transition pore (MPTP) to regulate mitochondria-dependent apoptotic or necrotic cell death [23–25]. Importantly, ROS produced from the mitochondria and subsequent oxidative damage are closely associated with the MPTP [26] and subsequent apoptosis [27]. Thus, inhibition of the MPTP by using cyclosporine A (CsA) to selectively inhibit CypD [28], rescues cell death in neuronal [29] and retinal [30] cell models.

The impetus for this study was the observation that QC-induced cell death, which was associated with mitochondrial localization of p53, was produced by QC-induced mitochondrial ROS and consequently lower mitochondrial membrane permeability (MMP) specifically in hESCs. Most importantly, using CsA to inhibit mitochondrial CypD, the expression of which was significantly high in hESCs, decreased the QC-mediated loss of MMP and mitochondrial cell death.

## RESULTS

### ROS production in hESCs by QC treatment

**Figure 1:**
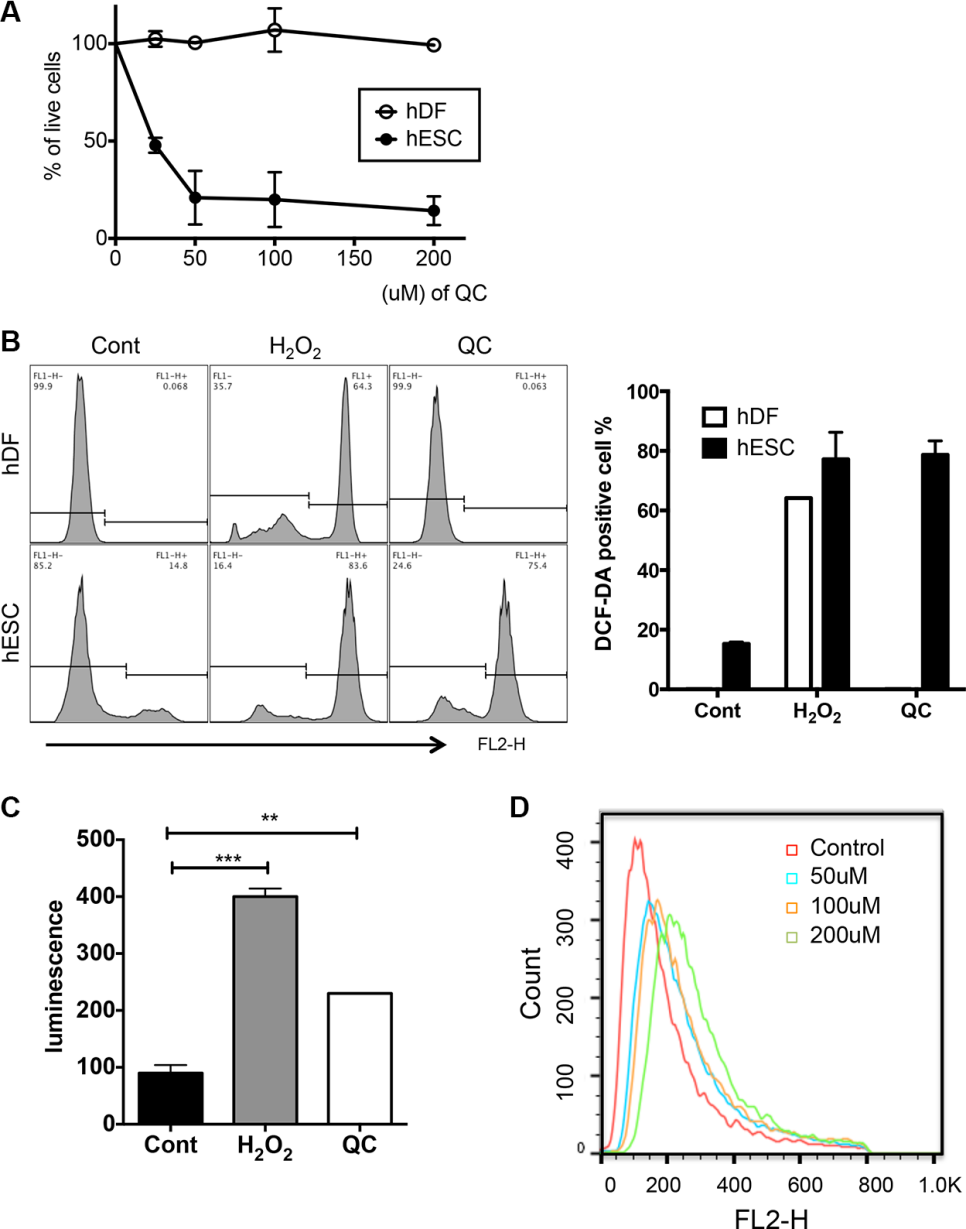
QC produces ROS in hESCs (**A**) Live cell population (dual negative population for Annexin V and 7-AAD) after indicated dose of QC treatment in hDFs and hESCs (**B**) After 2 hours treatment with H_2_O_2_ or QC, hDFs (upper panels) or hESCs (lower panels) were stained with DCF-DA and ROS level was determined by flow cytometry. The relative level of DCF-DA positive population was shown as a bar graph (right panel). (**C**) hESCs were incubated with lucigenin with H_2_O_2_ or QC for 20 minutes. Relative level of ROS measured by luminometer was shown as bar graph. (**D**) Mitochondrial ROS production was determined by MitoSOX staining and following flow cytometry. The results represent one of the experiments performed twice.

Consistent with our previous studies [6], QC treatment induced marked cell death only of hESCs and not of human dermal fibroblasts (hDFs) in a dose-dependent manner (Figures 1A, S1A and S1B). Given that QC acts either as a pro-oxidant to be cytotoxic to cancer cells with active proliferation [8, 10] or as an anti-oxidant [9], depending on the cell models, we surmised that QC may serve as a pro-oxidant to produce ROS in hESCs, which undergo active proliferation similar to that in cancer cells. To test this idea, we first compared the level of ROS in hESCs with that in hDFs, which were used as a model for differentiated cells and which are highly resistant to QC-induced cell death (Figure 1A). After 2 hours of QC treatment, ROS production was observed and maximized in hESCs but not hDFs, whereas H_2_O_2_ treatment produced similar levels of ROS in hESCs and hDFs (Figures 1B and S1C). Consistently, QC-induced ROS production in hESCs was also confirmed by a lucigenin assay (Figure 1C) [31]. Among diverse sources for ROS production, based on the increase in positive MitoSOX staining in hESCs induced in a dose-dependent manner by QC treatment, mitochondrial superoxide production [32] was concurrently increased with QC-induced cell death (Figure 1D). It is of note that as shown previously in mouse ESCs [14], Sestrin 2 (SESN2), an anti-oxidant gene, of which expression occurs in p53 dependent manner was not induced by QC treatment unlike hDFs (Figure S1D).

### ROS are responsible for QC-induced cell death in hESCs

**Figure 2:**
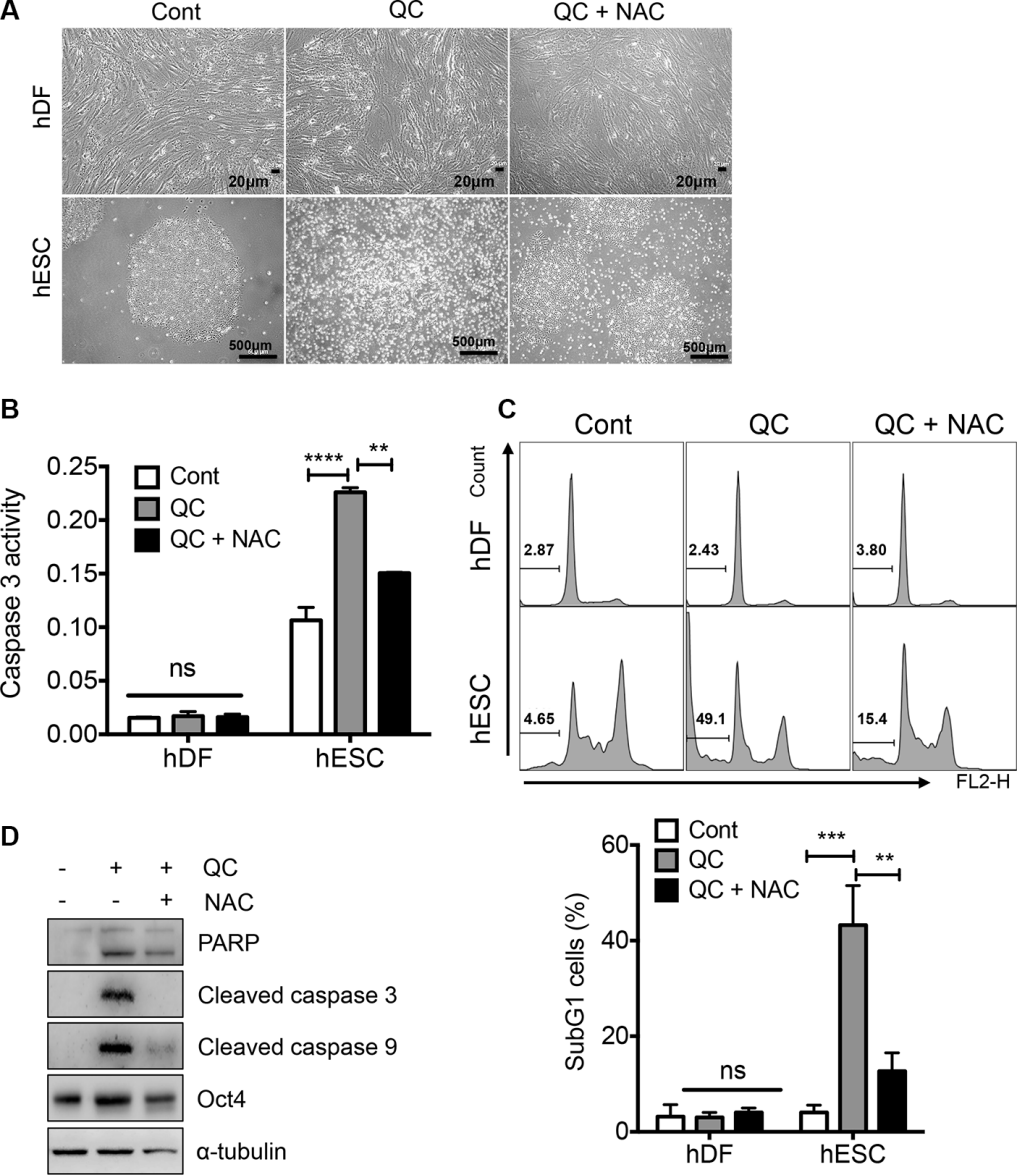
ROS is responsible for QC induced cell death in hESCs (**A**) 1 mM of NAC was pretreated 2 hours prior to 50 μM of QC treatment. Light microscopic images for hDFs (upper panels) and hESCs (lower panels) were shown [scale bar = 20 µm (top) or 500 µm(bottom)]o. (**B**) Relative level of caspase-3 activity of hDFs and hESCs after QC treatment was shown in a bar graph (*n* = 3). (**C**) Flow cytometry plots for sub-G1 population of hDF or hESCs 16 hours after 50 μM of QC treatment in the presence or absence of 1mM of NAC were shown (top panels). Sub-G1 population was represented by a bar graph (bottom panel). (**D**) Immunoblotting analysis for indicated protein level was shown. α-tubulin was used for equal loading control.

It has been shown that ESCs are highly sensitive to oxidative stress and readily undergo cell death [14]. In addition, mitochondrial cell death is highly active in hESCs due to ‘mitochondrial priming’ [20], p53 mitochondrial translocation [6] or high expression of active BAX at the Golgi complex [19]. Considering the fact that oxidative insult toward the mitochondria in hESCs promotes strong cell death [2], we surmised that mitochondrial superoxide production (Figure 1D) would be important for QC-induced cell death in hESCs (Figure 1A). To test this idea, we applied N-acetyl cysteine (NAC), a well-known antioxidant, prior to QC treatment and then monitored cell death. As predicted, inhibition of mitochondrial superoxide production (determined using MitoSOX) by NAC pretreatment (Figure S2), significantly reduced the typical morphological changes associated with cell death in hESCs, whereas hDFs remained unaltered regardless of QC treatment (Figure 2A). Consistently, NAC pretreatment lowered the QC-induced caspase-3 activity (Figure 2B) and sub-G1 population (Figure 2C) in hESCs. Additionally, the increased caspase-9 activation and subsequent caspase-3 activation induced by QC treatment, which are responsible for mitochondrial dependent cell death, were partly rescued by NAC pretreatment (Figure 2D). Collectively, these data strongly suggest that mitochondrial ROS serve an important role in regulating QC-mediated cell death in hESCs.

### Quercetin induces p53 mitochondrial translocation

**Figure 3:**
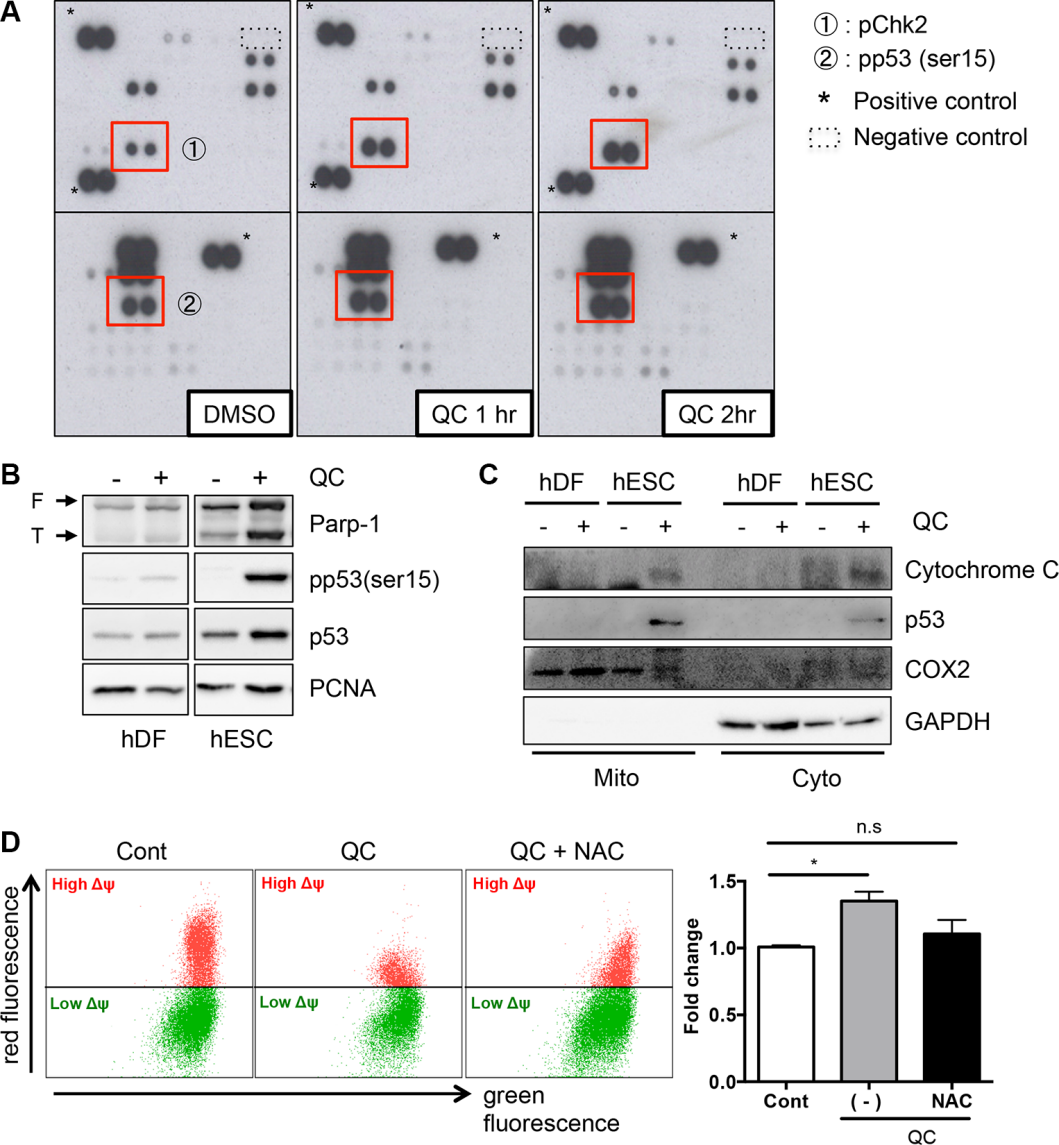
QC induces p53 mitochondrial translocation (**A**) hESCs protein lysate at indicative time after QC treatment was subjected to human phospho-kinase array. The red boxes indicate Chk2 phosphorylation on Thr68 (indicated with ①) and p53 phosphorylation on Ser15 (indicated with ②) respectively. (**B**) hESCs protein lysate was determined by immunoblotting analysis with indicative antibodies. α-tubulin was used as loading control. (**C**) Undifferentiated hESCs and hDFs were fractionated into mitochondrial (Mito) and cytoplasmic (Cyto) fractions, 12 hours after QC treatment. The level of p53 in indicated fractions was determined by immunoblotting. The typical marker proteins of all fractions such as GAPDH for cytoplasm and COX2 for mitochondria were used. (**D**) hESCs, pretreated with 1 mM of NAC 1 hour prior to QC treatment, was subjected to 1 μM of JC-1 staining for 30 minutes and followed by flow cytometry.

To measure downstream effects of mitochondrial cell death by QC-induced ROS in hESC, cells were treated with QC, and their lysates were then subjected to a Human Phospho-Kinase Array kit (Figure 3A). The 43 antibodies in the kit detect phosphorylation events that are known to play key roles in cell signaling, including phosphorylation of checkpoint kinase 2 (Chk2) on Thr68 and of p53 on Ser15, which were clearly enhanced in a time-dependent manner. First, we evaluated the phosphorylation of Chk2, which acts as an upstream kinase for p53. Chk2 phosphorylation gradually increased in QC-treated hESCs in a time-dependent manner (Figure S3A). Unexpectedly, however, attenuation of Chk2 phosphorylation (pChk2) by KU-55933, a chemical inhibitor of Ataxia telangiectasia mutated (ATM) (an upstream kinase for Chk2), could not rescue QC-mediated cell death of hESCs (Figure S3B). Thus, we ruled out a role for Chk2 activation and then examined p53 in QC-induced cell death because the phosphorylation of p53 and consequent p53 stabilization by QC treatment was more evident in hESCs but not in hDFs (Figure 3B). It is noteworthy that the ‘mitochondrial priming’ that indicates a high susceptibility to mitochondrial cell death occurs by cytoplasmic p53 [20]. Previously, we also showed that QC-induced cell death in hESCs could be attributed to p53 mitochondrial translocation [6], which is sufficient to trigger mitochondrial cell death [33, 34]. Consistently, cytochrome c, which is released from mitochondria when the MMP is altered during mitochondrial cell death [35], was found in the cytoplasmic fraction after QC treatment of hESCs, when p53 was accumulated in the mitochondria (Figure 3C). In this context, depletion of p53 in hESCs was likely to weaken the cell death effect of QC (Figure S3C). These data strongly imply that mitochondrial p53 translocation in hESCs after QC treatment is involved in this process. Because NAC pretreatment along with QC lowered oxidative stress and prevented cells from losing MMP (Figure 3D), we surmised that the expression of a certain protein in the mitochondria of hESCs but not in hDFs might be involved in the sensitivity to QC-induced mitochondrial cell death.

### Cyclophilin D contributes to quercetin-induced cell death in hESCs

**Figure 4:**
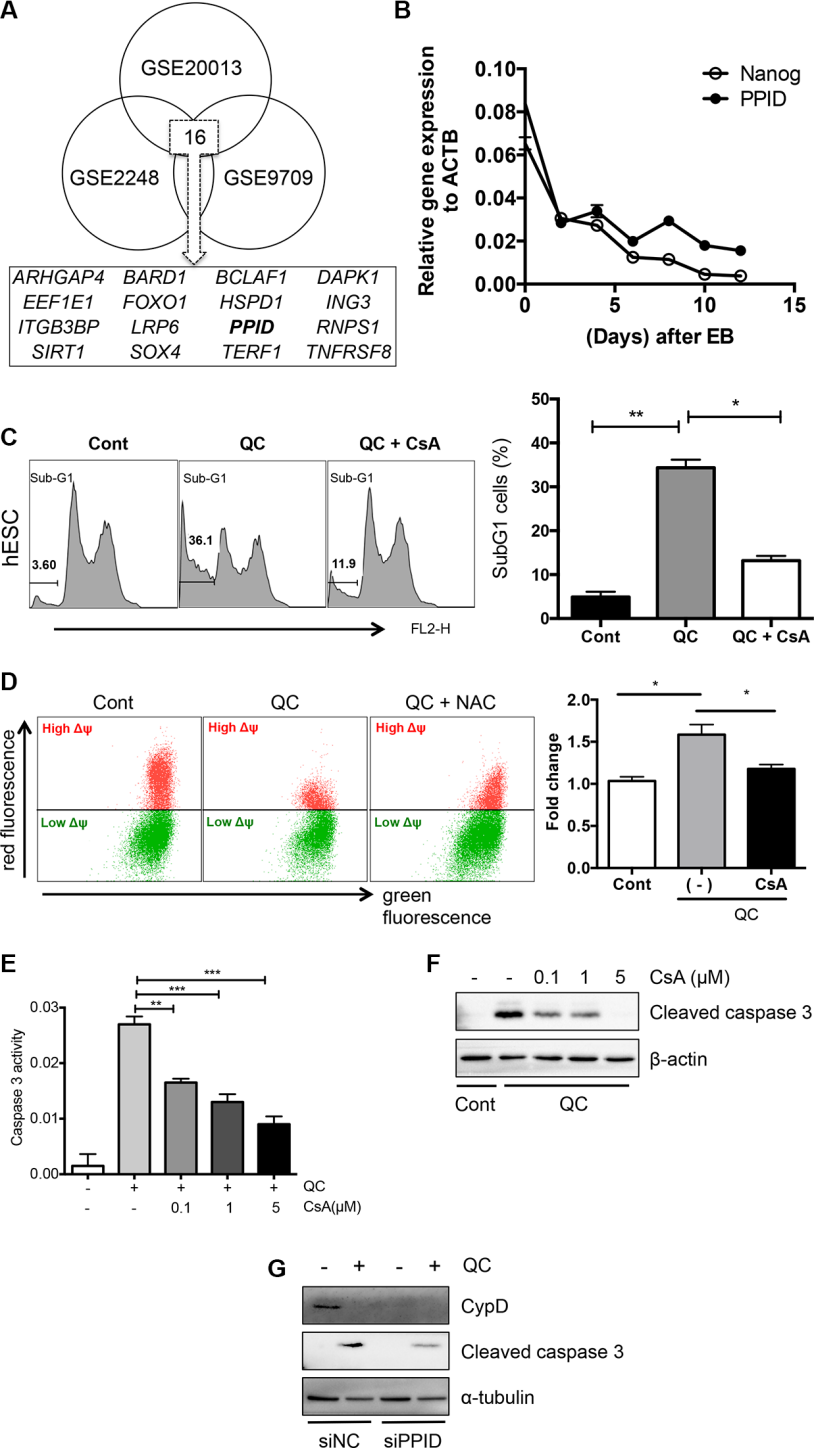
Cyclophilin D contributes to QC induced cell death of hESCs (**A**) GEO analysis to deduce candidate genes that regulate the mitochondrial cell death of hESCs compared to the differentiated counterparts. Three independent GSE studies including induced pluripotent stem cells (iPSCs) vs hDFs (GSE9709), hESCs vs human mesenchymal stem cells (hMSCs) (GSE2248) and hESCs vs endothelial cells (EC) (GSE20013) were used to select out common genes. (**B**) mRNA expression level of *NANOG* and *PPID* at indicated days during spontaneous differentiation of hESCs by real-time PCR analysis. (**C**) Sub-G1 population of hDF (upper panels) or hESCs (lower panels) 16 hours after 50 μM of QC treatment with or without 1 μM of cyclosporine A (CsA) pretreatment were shown. Sub-G1 population was represented by a bar graph on the left panel. (**D**) hESCs, pretreated with 1 μM of CsA for 1 hour were treated with QC for another 2 hours. In turn, mitochondrial membrane potential was subsequently analyzed by staining with 1 μM of JC-1 dye for 30 minutes. (**E**) Relative level of caspase-3 activity of hESCs 16 hours after 50 μM of QC treatemtn was compared with indicative dose of CsA pretreatment (*n* = 3). (**F**) Cell death was determined by immunoblotting for cleaved caspase-3. β-actin was used as a loading control. (**G**) hESCs, 48 hours after transfection with siRNA for control (siNC) or PPID (siPPID) were treated with QC. Protein levels were determined by immunoblotting analysis with indicative antibodies. α-tubulin was used for equal loading control.

**Figure 5:**
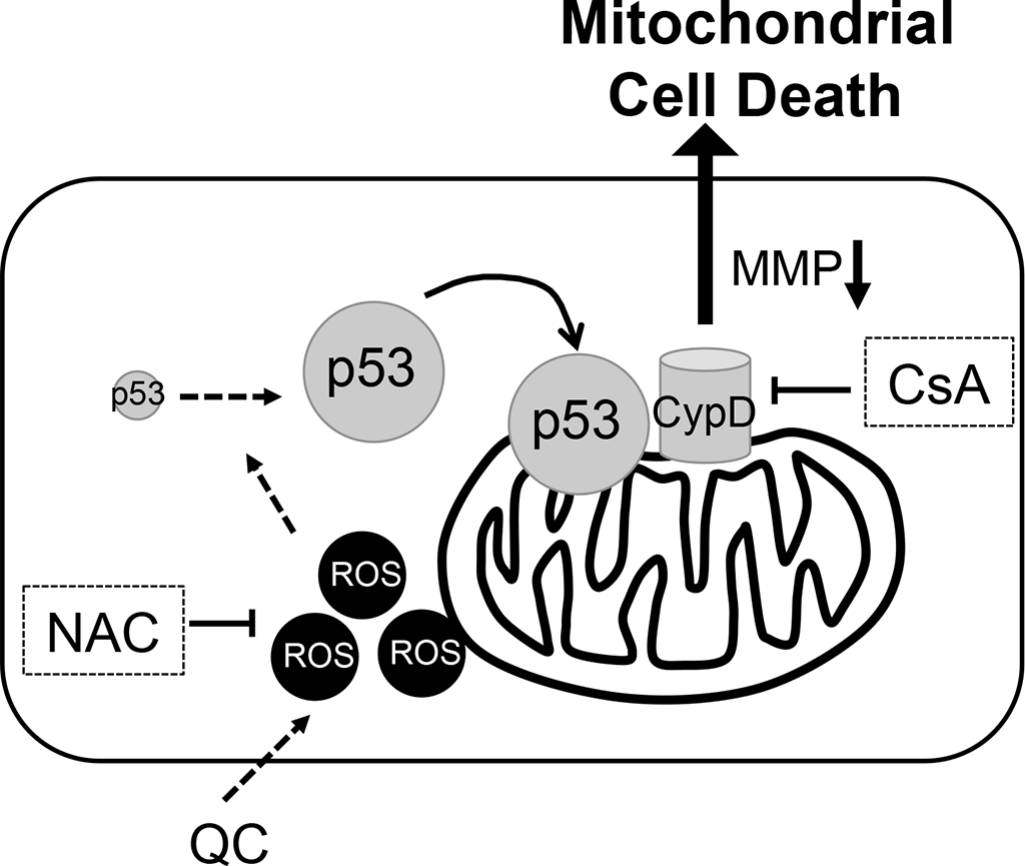
Proposed model of molecular event for QC induced hESCs cell death, Perpendicular line for inhibition, NAC for N-acetyl cysteine, MMP for mitochondrial membrane permeability, CypD for Cyclophilin D, and CsA for cyclosphorine A

Next, to identify an event downstream of the mitochondrial localization of p53 to release cytochrome c (Figure 3C) and lower MMP (Figure 3D), we used a gene expression omnibus (GEO) database search (http://www.ncbi.nlm.nih.gov/geo/) as described previously [36]. Three independent GSE datasets (GSE20013, GSE2248, and GSE9709), which were obtained from comparisons between human pluripotent stem cells and differentiated cells (Figure S4A), were selected to find commonly upregulated pro-apoptotic genes in hESCs. We narrowed down the gene list using the Gene Ontology (GO) processes “positive regulation of apoptotic process” and “apoptotic process” and the GO components “mitochondrion” and “cytoplasm”, and we identified 16 gene candidates (Figures 4A and S4B). Among these 16 candidates, we were particularly interested in death-associated protein kinase 1 (*DAPK1*), for which high expression in hESCs has been reported previously [6] and peptidylprolyl isomerase D (*PPID*, encoding the protein cyclophilin D (CypD)), the mitochondrial expression of which [23] is critical for inducing p53-dependent cell death under oxidative stress [37, 38]. Importantly, the CypD level in hESCs was much higher than that in hDFs (Figure S4C), and it was markedly decreased along with *NANOG* when hESCs underwent spontaneous differentiation (Figure 4B). Therefore, we hypothesized that mitochondrial expression of CypD, which was higher in hESCs than in hDFs and mesenchymal stem cell derived from hESCs (hESC-MSCs) [39] (Figure S4C and S4D), might be associated with QC-induced hESC cell death because increased CypD, results in cytochrome c release and subsequent caspase 9 activation along with increased TUNEL staining [23]. Moreover, we found that *PPID* gene expression was dramatically induced by QC treatment in hESCs but not hDFs (Figure S4E). Thereby, it is highly plausible that high CypD level in hESCs would be responsible for QC mediated cell death in hESCs. To investigate this possibility, we tested whether inhibition of CypD by CsA, which has been previously shown to inhibit cell death from the mitochondria [30, 38, 40], affected cell death in hESCs. It has been well described that CypD promotes mitochondrial permeability transition (MPT) [23], which is strongly associated with necrosis [25] or apoptosis [30, 41]. As predicted, CsA pretreatment significantly lowered QC-mediated hESC cell death (Figure 4C) and MMP (Figure 4D). Inhibition of the loss of MMP (Δψ loss) by pretreatment with CsA followed by treatment with QC (Figure 4D) resulted in decreased caspase-9 activation (Figure S4F). Additionally, caspase-3 activity is decreased by CsA in a dose-dependent manner (Figure 4E and 4F). To exclude the possible off-target effect of CsA, CypD was depleted by siRNA for *PPID* and cell death effect of QC was monitored. Consistently, loss of CypD expression by *PPID* knockdown (Figure S4G), inhibited cell death by QC treatment (Figure 4G). We also demonstrated that p53 interacted with CypD in hESCs as shown previously [37] (Figure S4H). Accordingly, we propose a model in which the increase in ROS production after QC treatment may stabilize p53 protein, which in turn translocates into the mitochondria. Mitochondrial p53 may interact with CypD to induce loss of MMP, which subsequently triggers mitochondrial cell death (Figure 5).

## DISCUSSION

Maintenance of genomic integrity of hESCs is critical because accumulated mutations can be transmitted to the cells subsequently derived from the hESCs at later stages of differentiation [42]. Thus, it is reasonable that hESCs develop efficient DNA repair machinery to repair a variety of DNA lesions caused by genotoxic stimuli including ROS and chemotherapeutic agents, as well as an active cell death mechanism to eliminate aberrant cells when the genomic insult is beyond their capacity to repair [43]. In particular, high susceptibility to genotoxic stress in hESCs results from a lower threshold for mitochondrial cell death [21], which is regulated by the cytoplasmic [20] or mitochondrial [6] p53 levels.

Previously, we demonstrated the high expression of pro-apoptotic genes including death-associated protein kinase 1 (*DAPK1*) (Figure 4A) and a few anti-apoptotic genes such as *BIRC5* (encoding Survivin) in undifferentiated hESCs [6]. Inhibition of *BIRC5* expression in hESCs with QC or with YM155, a Survivin inhibitor, is sufficient to induce cell death selectively in hESCs [6] (Figure 1A). Importantly, treatment with QC stabilizes p53 and induces mitochondrial translocation to trigger mitochondrial cell death [6]. However, how p53 is stabilized by QC treatment has not been fully addressed. Herein, we demonstrated that treatment of QC increased ROS in hESCs, mostly from the mitochondria (Figure 1), where QC-induced cell death was triggered by lower MMP (Figure 3D). We also revealed that stabilized p53 in hESCs triggered cell death by mitochondrial translocation (Figures 4C and S3C). Most importantly, p53-dependent mitochondrial cell death was closely associated with CypD expression in the mitochondria, as inhibition of CypD by CsA or loss of CypD significantly lowered the rates of mitochondrial cell death and loss of MMP (Figure 4).

Still, how QC treatment increased ROS production only in hESCs and not in hDFs is not entirely clear (Figure 1). QC can act either as a pro-oxidant or as an anti-oxidant in a cell-type-dependent manner, and most importantly, the cytotoxic effect of QC as a pro-oxidant is most evident in aggressive cancer cells [10]. One of the properties shared by aggressive cancer cells and hESCs is mitochondrial dysfunction, which is represented as highly mutated mitochondrial DNA and skewed oxidative phosphorylation [44]. Most of all, the failure to induce p53-dependent anti-oxidant gene such as *SESN2* in hESCs under oxidative stress (Figure S1D) might potentiate QC-induced oxidative stress and allow it to reach the threshold for mitochondrial cell death. An interesting possibility for a subsequent study is the hypothesis that attenuated mitochondria in both aggressive cancer and hESCs could be attributed to QC-dependent mitochondrial ROS production, leading to mitochondrial cell death. In addition, we found that CypD protein level was rather markedly decreased after QC treatment (Figure 4G) although *PPID* gene expression was highly induced (Figure S4E). On the basis of these results, we surmised that QC treatment promptly destabilized CypD after inducing cell death. It is remained unanswered how CyPD protein level was quickly downregualted by QC treatment.

In this study, we present evidence that ROS production by QC to stabilize p53 in the mitochondria contributed to QC-mediated hESC cell death. The high expression of CypD in hESCs, compared to hDFs was associated with mitochondrial cell death by QC treatment. This study provides important insight into the hESC-specific mitochondrial cell death machinery.

## MATERIALS AND METHODS

### Cell culture and treatment

Human ESCs (H9) was maintained in mTeSR-E8 medium on matrigel coated 60 mm dishes. Human dermal fibroblasts (hDF) were maintained in high-glucose DMEM (Gibco, cat#. 11995) supplemented with 10% (vol/vol) Fetal Bovine Serum (Gibco, cat#. 16000), 1% GlutaMAX (Gibco, cat#. 35050), 1% nonessential amino acids (Gibco, cat#. 11140) and 0.1% gentamycin (Gibco, cat#. 15750). For QC treatment, hESCs were cultured in ESC medium without β-mercaptoethanol to maximize the ROS effect on cell death (Gibco, cat#. 21985). ESC medium was composed in DMEM/F12 supplemented with 20% (vol/vol) Knock-Out Serum Replacement (Gibco, cat#. 12618) with 0.1% gentamycin, 1% nonessential amino acids and 4 ng/ml bFGF2. For ROS inhibition, hESCs and hDFs were pre-treated with NAC 3 mM for 2 hours and washed off with PBS. QC 100 μM was treated subsequently. For CypD inhibition, hESCs and hDFs were pre-treated with CsA 1 μM for 1 hour and added QC 100 μM subsequently.

### Reagents and antibodies

Quercetin (cat#. Q0125) and N-acetyl-L-cysteine (cat#. A7250) were purchased from Sigma-Aldrich. KU-55933 (cat#. 1685–5) was purchased from Biovision. Cyclosporin A (cat#. 239835) was purchased from Merk Millipore. Antibodies used in the present study; anti Parp-1 (sc-1750), ERK2 (sc-154), p53 (sc-126), PCNA (sc-56), COX2 (sc-746), α–tubulin (sc-8035), β-actin (sc-47778) were purchased from Santa Cruz Biotechnology. Oct-4 (2840P), cleaved caspase-3 (9664S), cleaved caspase-9 (#7237), pp53 ser15 (#9286), Acetyl p53 (#2525), pChk2 (#2661) were purchased from Cell Signaling. Cytochrome C (05–479) was purchased from Merck Millipore.

### FACS analysis

For all of the FACS analysis, FACS calibur (BD Biosciences) and Flowjo software were used. Cells were stained using 1 μg /ml propidium iodine for 1 hour (PI) in the presence of 500 μg /ml RNase.

### Mitochondrial isolation

Mitochondria and cytosol fractions were separated according to the instructions included with the Q-proteome Mitochondria Isolation Kit (Qiagen, cat#. 37612).

### Detection of intracellular ROS

After 2 hours from Quercetin treatment, ROS levels were determined by incubating cells with 20 μM dichlorfluorescein diacetaate (DCF-DA, Abcam) or 5 μM mitoSOX (Invitrogen, cat#. M36008) for 30 min at 37°C. We washed the cells twice in PBS, trypsinized them and measured fluorescence with a FACs analysis. (Excitation at 488 nm, emission at 515–545 nm for DCF-DA and excitation at 510 nm, emission at 580 nm for mitoSOX). Data were analyzed with Flowjo.

### Statistical analysis

The graphical data were presented, as mean ± SEM. Statistical significance among three groups and between groups was determined using one-way or two-way ANOVA after Bonferroni posttest and Student t test, respectively. Significance was assumed for *P* < 0.05 (*), *P* < 0.01 (**) and *P* < 0.001 (***).

## SUPPLEMENTARY MATERIALS FIGURES


