# Supplementary material for: Quercetin induced ROS production triggers mitochondrial cell death of human embryonic stem cells
Source: Oncotarget. 2016 Aug 5;8(39):64964–73. doi: 10.18632/oncotarget.11070 (PMC5630304; doi:10.18632/oncotarget.11070)
Supplement: Supplementary file 1 [file oncotarget-08-64964-s001.pdf]

Quercetin induced ROS production triggers mitochondrial cell death of human embryonic stem cells

Supplementary Materials

SUPPLEMENTARY MATERIALS AND METHODS

RNA Isolation and Quantitative Real-Time PCR Analysis

Total RNA was extracted from cells using Trizol Reagent (Invitrogen). By adding chloroform, Trizol was removed and mRNA was precipitated by isopropanol. The RNA precipitates were washed by 75% ethanol. The amount and purity of RNA were measured by detecting the optical density at 260 nm and 280 nm with a UV spectrometer. The amplified template was detected using SYBR® Premix Ex TaqTM (Takara Bio Inc.) with a real-time PCR system (Roche, LightCycler® 480). PCR was conducted in 20 µl of reaction volume containing 10 µl SYBR green mix (Applied Biosystems), 0.1 µM of each primer, and 2 µl of cDNA template with the following conditions: 95°C for 30 sec for denaturation of template, and 40 cycles of 95°C for 5 sec, 58°C for 15 sec, and 72°C for 20 sec. The average threshold cycle for each gene was determined from triplicate reactions, and the levels of gene expression relative to GAPDH were determined. Gene specific primers were follows: β-actin (5-GTCCTCTCCCAAGTCCACAC-3, 5-GGGA GACCAAAAGCCTTCAT-3); hNanog (5- AAATT GGTGATGAAGATGTATTCG-3, 5-GCAAAACAG AGCCAAAAACG-3); hPPID (5- TCCAGATTTC CCTGAGGATG-3, 5-GGTTGCAGCTTGGCTCT ATC-3).

Immunoblotting

Cells were harvested with trypsin and washed twice with phosphate-buffered saline (PBS). After washing, cells were lysed with 50 µl of tissue lysis buffer and centrifuged at 13,000 rpm for 10 minutes to clarify lysates. Approximately 20 µg of total proteins were separated by SDS-PAGE and transferred to PVDF membrane. The membrane was blocked with 5% nonfat dry milk in tris-buffered Saline (TBS) with 0.1% Tween-20 (TBS-T) for 1 hour and probed with appropriate primary antibodies hours. The membranes were washed several times in TBS-T and incubated with horseradish peroxidase-conjugated secondary antibodies (0.1 µg/ml; Jackson Immunoresearch Laboratories).

Caspase-3 activity assay

The Ac-DEVD-AMC caspase-3 fluorogenic substrate (BD Pharmingen) was used for assays performed according to the manufacturer's instructions.

Phospho-kinase array

Relative levels of protein phosphorylation were determined according to the instructions with human phospho-kinase array kit (R&D Systems, cat#. ARY003B).

JC-1 staining

Mitochondrial membrane potential was measured using the JC-1 Mitochondrial Membrane Potential Array Kit (ab113850, Abcam) following the manufacturer's instructions.

Co-immunoprecipitation

Approximately 500 µg of total proteins were incubated with 1 µg of CypD antibodies (cat# sc-137157) at 4°C overnight [IgG (cat# sc-2025) only for negative control]., followed by addition of protein G Sepharose (17-0618-01, GE health) beads, and then incubated at 4°C for an additional 4 hours. The precipitates were washed with tissue lysis buffer for each 20 minutes three times, and then separated by SDS-PAGE. Immunoblotting was conducted using standard methods. For avoiding interference from the IgG heavy chain during immunoblotting, VeriBlot (cat# ab131368, Abcam) was used for secondary antibody.

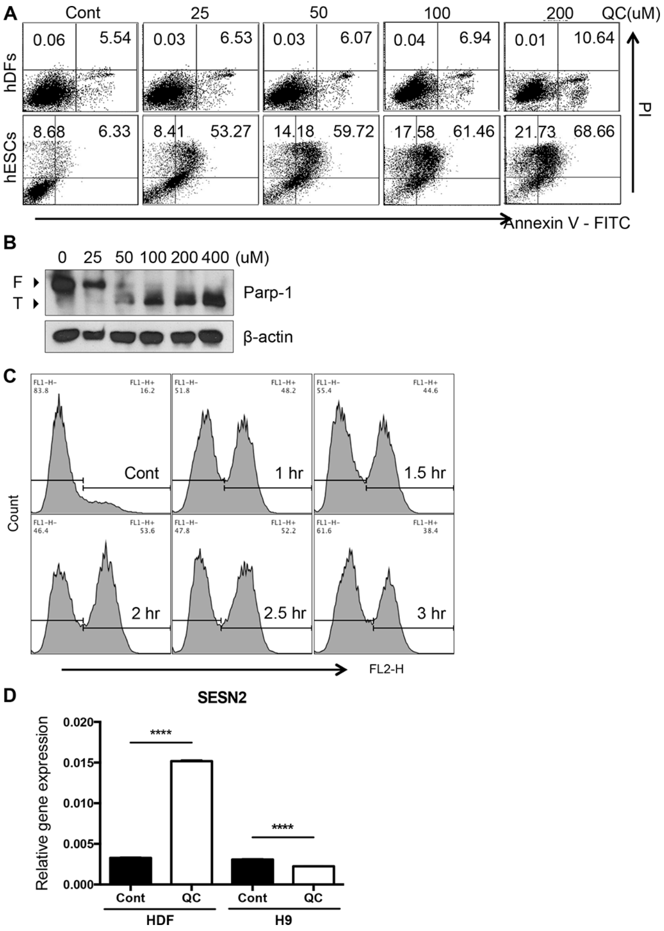

Supplementary Figure S1: (**A**) The dot plot images of hDFs and hESCs after indicated dose of QC treatment determined by FACS analysis for Annexin V and 7-AAD staining. (**B**) hESCs were treated with indicative dose of QC for 16 hours. Cell death was determined by immunoblotting for Parp-1. b-actin for equal protein loading (F: full length, T: truncated Parp-1) (**C**) The ROS level in hESCs was measured by DCF-DA staining at indicated time after QC treatment (50 μM). (**D**) mRNA expression level of antioxidant enzyme, SESN2 was measured 24 hours after QC treatment.

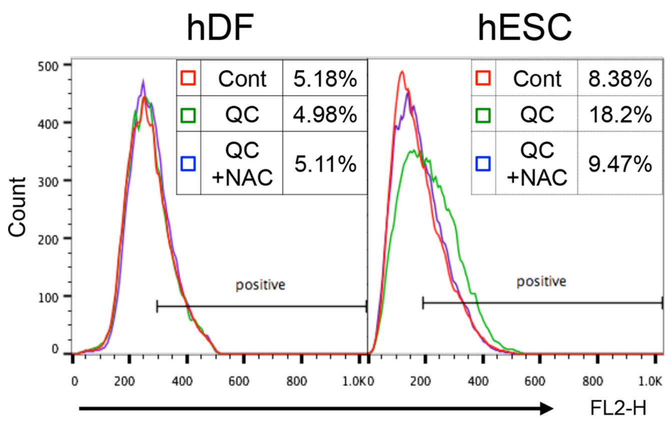

Supplementary Figure S2: hDFs and hESCs were stained with MitoSOX 2 hours after QC treatment (50 μM) and MitoSOX positive population was determined by flow cytometry.

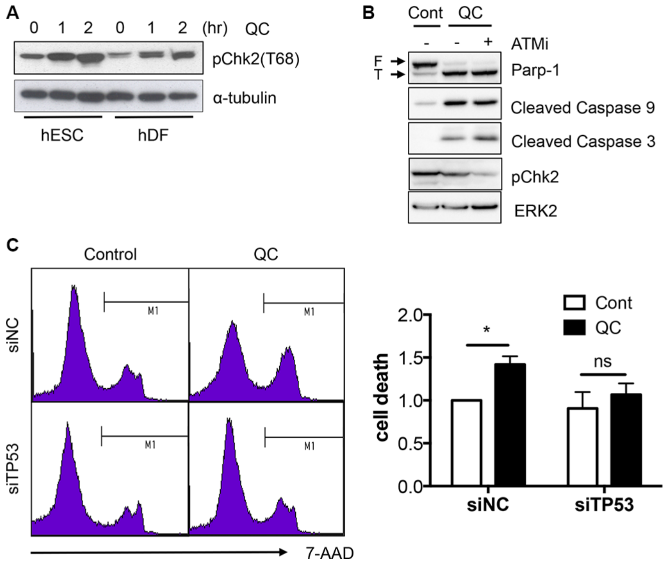

Supplementary Figure S3: (**A**) Protein lysate from hESCs or hDF at indicative time after QC treatment (50 μM) was determined by immunoblotting analysis. a-tubulin for equal protein loading, (**B**) 30 μM of KU-55933 was pretreated 1 hour prior to QC treatment to inhibit ATM activity. Cell death was determined by immunoblotting analysis for cleaved caspase-3, -9 and Parp-1. ERK2 for equal protein loading control, (**C**) Cell death of hESCs 16 hours after 50 mM of QC treatment with or without p53 expression with siRNA for p53 was determined by 7-AAD staining and followed by flow cytometry.

A

GSE number	Cell line and Sample Size	Reference (PMID#)
GSE9709	iPSC (8) and Human Neonatal dermal fibroblast (2)	19383391
GSE2248	hESC (3) and hMSC (3)	15971941
GSE20013	hESC (4) and hESC derived EC (4)	10700174

B

Gene Symbol	Gene Title	Gene ID
ARHGAP4	Rho GTPase activating protein 4	393
BARD1	BRCA1 associated RING domain 1	580
BCLAF1	BCL2-Associated Transcription Factor 1	9774
DAPK1	death-associated protein kinase 1	1612
EEF1E1	eukaryotic translation elongation factor 1 epsilon 1	9521
FOXO1	forkhead box O1	2308
HSPD1	heat shock 60kDa protein 1 (chaperonin)	3329
ING3	inhibitor of growth family, member 3	54556
ITGB3BP	integrin beta 3 binding protein (beta3-endonexin)	23421
LRP6	low density lipoprotein receptor-related protein 6	4040
PPID	peptidylprolyl isomerase D	5481
RNPS1	RNA binding protein S1, serine-rich domain	10921
SIRT1	sirtuin 1	23411
SOX4	SRY (sex determining region Y)-box 4	6659
TERF1	telomeric repeat binding factor (NIMA-interacting) 1	7013
TNFRSF8	tumor necrosis factor receptor superfamily, member 8	943

C

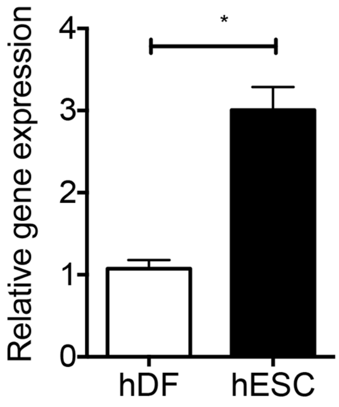

D

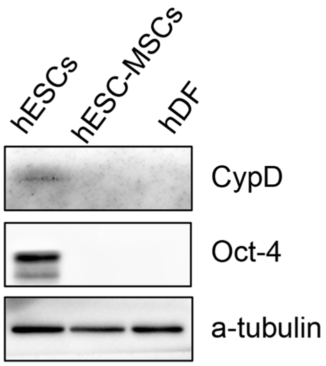

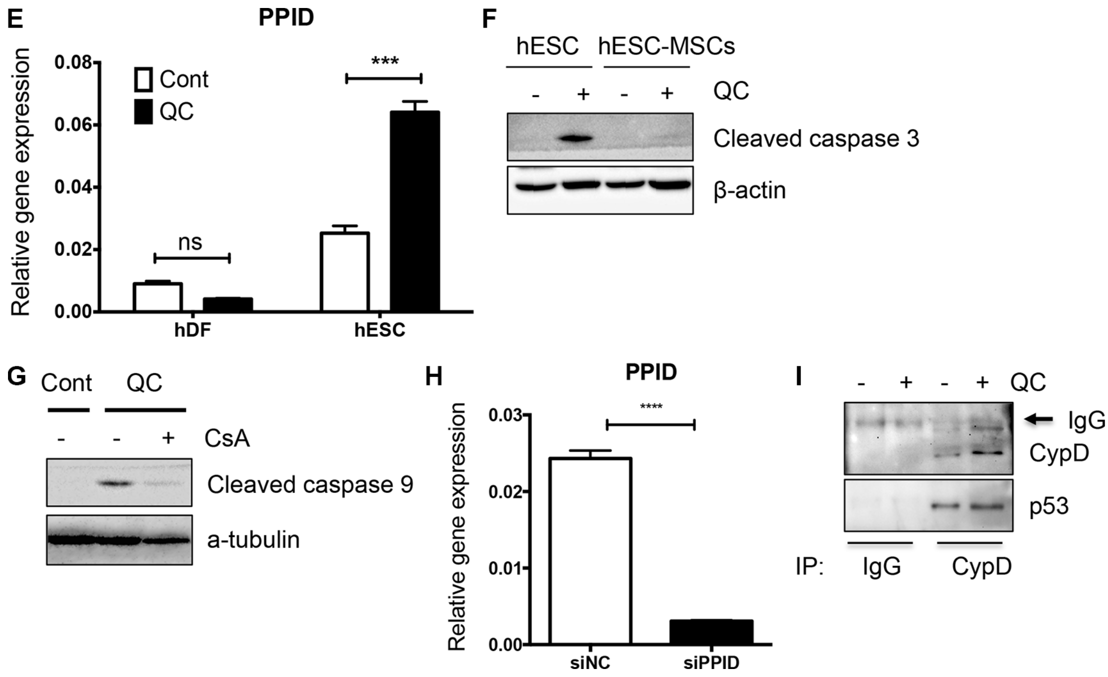

Supplementary Figure S4: (A) The information about GSE studies used in this study. (**B**) Gene symbol, Gene title and Gene ID about selected genes are shown. (**C**) Relative basal mRNA level of *PPID* in hDFs and hESCs was determined by real-time PCR analysis. (**D**) Basal protein level of CypD was measured in hESCs, hESC derived MSCs and hDFs by immunobloting analysis. (**E**) The effect of QC on *PPID* gene expression was measured using real-time PCR analysis. (**F**) Cell death was determined by immuneblotting with hESC and hESC derived MSCs (hESC-MSCs) lysates. β-actin was used as a loading control. (**G**) Immunoblotting analysis of cleaved caspase-9 was shown with a-tubulin as loading control. (**H**) Efficient knockdown of *PPID* in hESCs is shown in mRNA level. (I) hESCs were treated QC for 16 hours. Immunoprecipitation with anti-CypD antibody was performed, followed by immunoblotting analysis for p53.
